# Rapid control of pandemic H1N1 influenza by targeting NKT-cells

**DOI:** 10.1038/srep37999

**Published:** 2016-11-29

**Authors:** Bianca L. Artiaga, Guan Yang, Tarun E. Hutchinson, Julia C. Loeb, Jürgen A. Richt, John A. Lednicky, Shahram Salek-Ardakani, John P. Driver

**Affiliations:** 1 Department of Animal Science, University of Florida, Gainesville, FL, USA; 2 Department of Pathology, Immunology, and Laboratory Medicine, University of Florida, Gainesville, FL, USA; 3 Emerging Pathogens Institute, University of Florida, Gainesville, FL, USA; 4 Diagnostic Medicine and Pathobiology and Center of Excellence for Emerging and Zoonotic Animal Diseases (CEEZAD), College of Veterinary Medicine, Kansas State University, Manhattan, KS, USA; 5 Department of Environmental and Global Health, University of Florida, Gainesville, FL, USA

## Abstract

Swine influenza A viruses (IAV) are a major cause of respiratory disease in pigs and humans. Currently approved anti-influenza therapies directly target the virus, but these approaches are losing effectiveness as new viral strains quickly develop drug resistance. To over come this challenge, there is an urgent need for more effective antiviral drugs. Here we tested the anti-influenza efficacy of the invariant natural killer T (NKT) cell superagonist, α-galactosylceramide (α-GalCer), which stimulates a wide array of anti-viral immune responses. We show that intranasal but not systemic administration of α-GalCer to piglets infected with pandemic A/California/04/2009 (CA04) H1N1 IAV ameliorated disease symptoms and resulted in the restoration of weight gain to the level of uninfected pigs. Correspondingly, viral titers in the upper-and lower-respiratory tract were reduced only in piglets that had received intranasal α-GalCer. Most significantly, lung inflammation as a consequence of virus persistence was largely prevented when NKT-cells were targeted via the respiratory route. Thus, targeting mucosal NKT-cells may provide a novel and potent platform for improving the course of disease in swine infected with seasonal and pandemic influenza viruses, and leads to the suggestion that this may also be true in humans and therefore deserves further study.

Swine influenza (SI) is an acute respiratory disease of pigs caused by three subtypes of influenza A viruses (IAV), H1N1, H1N2, and H3N2^1^. Infections often lead to additional respiratory diseases, resulting in substantial economic losses for pork producers^2^. The risk of SI outbreaks continues to increase due to the accelerating pace at which swine IAVs are evolving^1^. Besides implementing strict biosecurity measures, vaccination is the most effective strategy to prevent SI infections. However, current swine influenza vaccines do not provide protective immunity for different IAVs even within the same virus subtype and there is often insufficient time to produce and distribute vaccines against emerging strains^3^,^4^. These shortcomings contribute to why millions of pigs continue to become infected with swine IAVs every year. Unfortunately, there are no available treatments for pigs already infected with IAVs, which is greatly needed for limiting IAV transmission and reducing production losses.

Of note, swine are susceptible to both human and avian influenza viruses and serve as a mixing vessel for the reassortment of novel viruses with the capacity to cause human pandemics^5^. Indeed, the 2009 H1N1 pandemic arose from a North American triple reassortant virus with genes derived from bird, swine and human flu viruses which further reassorted with a Eurasian pig flu virus^5^. The pandemic resulted in millions of hospitalizations and an estimated 300,000 deaths^6^. Although swine influenza is typically caused by only three subtypes of influenza A viruses, these continue to evolve at an ever increasing pace; in combination with the possibility of the introduction of a novel subtype into pigs, this raises the likelihood of more frequent pandemics in future.

The current leading strategy for controlling the spread of influenza and treating infected humans relies on administering neuraminidase (NA) inhibitors (NAI) as antiviral therapies. However, a major concern with NAIs is the evolution of virus mutants that develop drug resistance^7^,^8^,^9^,^10^,^11^. Moreover, to be effective, treatment with current NAIs such as Tamiflu® must occur within hours after symptoms develop for any effect. Other significant deficiencies include that they generally have a limited benefit for relieving flu symptoms, preventing infection, transmission or complications and they typically induce a relatively high rate of adverse drug reactions^12^,^13^. These shortcomings along with the low coverage of current vaccines require the urgent development of more effective approaches to counter future influenza outbreaks. One approach may be through targeting innate immune cells that can rapidly inhibit IAV replication and which are more refractory to resistance mechanisms of IAVs.

Invariant natural killer T (NKT) cells are a highly specialized subset of immune cells that share phenotypic and functional characteristics of both innate NK cells and adaptive T lymphocytes. These cells express a semi-invariant T cell receptor (TCR) that recognizes self and foreign glycolipid antigens presented by the non-polymorphic CD1d molecules on antigen presenting cells. Once activated, NKT-cells stimulate a diverse array of innate and adaptive immune responses important for controlling a wide range of infectious diseases^14^. We recently demonstrated that swine co-injected with the NKT-cell superagonist, α-galactosylceramide (α-GalCer), and a model antigen hen-egg lysozyme (HEL) generate strong antigen-specific T cell and antibody responses^15^. We also demonstrated that intramuscular (i.m.) immunization of UV-killed whole influenza virus pandemic H1N1 (pH1N1) (A/California/04/2009 [kCA04]) and α-GalCer is a safe and effective means of protecting against a homologous challenge with live virus^16^.

In the current work we extend these studies by showing that delivery of α-GalCer into the nasal passages of piglets infected with pH1N1 generates immunity that effectively inhibits viral replication in airway tissues and markedly decreases virus shedding. Most significantly, administration of α-GalCer to IAV-infected animals reduced disease severity and lung pathology, and did not cause any adverse immune reactions in young piglets. Our results demonstrate the possibility of using NKT-cell agonists to reduce the severity of influenza infections in swine, thereby limiting IAV transmission from pigs to humans. They also show potential for harnessing NKT-cell agonists for treating IAV infections in humans for which effective therapies are currently lacking.

## Results

### α-GalCer reduces flu symptoms and restores weight gain in influenza-infected pigs

To determine whether α-GalCer mitigates influenza infections in swine, we compared the efficacy of α-GalCer delivered by the intranasal (i.n.) or i.m. routes for treating IAV-infected piglets. Accordingly, three-week-old commercial breed piglets were infected with a large inoculum (5 × 10^5^ TCID_50_) of pandemic H1N1 (pH1N1) A/California/04/2009 (CA04) virus delivered directly into the lungs by intratracheal (i.t.) inoculation. At the same time, pigs were treated with either vehicle (Mock/CA04) or with 100 μg/kg α-GalCer injected into the neck muscle (IM αGC/CA04), or dripped into the nostrils (IN αGC/CA04). As a control, another group of pigs were mock infected and sham treated with vehicle (Mock/Mock). Characteristics of pigs in different treatment groups are described in Table 1.

**Figure 1:**
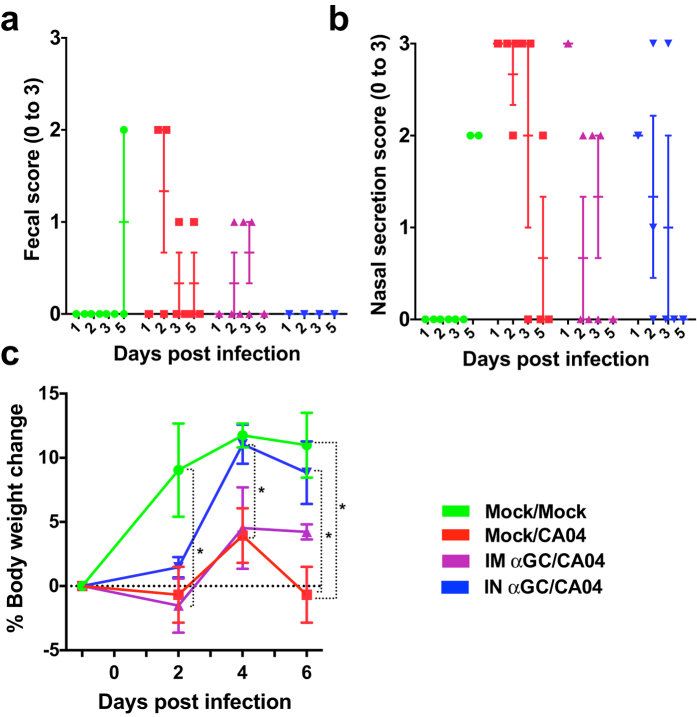
Intranasal α-GalCer administration reduces flu symptoms and restores body weight gain in IAV-infected pigs. (**a,b**) Health scores reported for individual pigs and mean ± standard error of the mean (SEM) for each treatment group on days 1, 2, 3 and 5 post infection (p.i.). (**a**) Fecal score: ranked from 0 to 3; 0: normal; 1: semi-solid; 2: liquid; 3: watery, presence of mucous or blood. (**b**) Nasal secretion: observed to be clear and serosa for all animals; ranked from 0 to 3, as 0: absent; 1: small volume discharge, from one nostril; 2: small volume, both nostrils; 3: large volume, both nostrils. (**c**) Percentage body weight gain compared to day −1 p.i. for SI virus-challenged pigs mock-treated with MEM (Mock/CA04) or treated with 100 μg/kg α-GalCer administered i.m. (IM αGC/CA04) or i.n. (IN αGC/CA04). Control pigs were mock challenged and mock-treated with MEM (Mock/Mock). Differences in weight gain were analyzed using the Kruskal-Wallis test. *P < *0.05*. Data are represented as mean ± SEM.

IN αGC/CA04 pigs did not develop diarrhea (0/3), in contrast to Mock/CA04 pigs (3/3) and IM αGC/CA04 pigs (2/3) (Fig. 1a). IN αGC/CA04 pigs also had reduced nasal discharge (Fig. 1b) and were more alert during the acute phase of infection (1–3 days post-infection [p.i.]) than pigs in the other CA04-infected groups. We observed some nasal discharge and diarrhea in control pigs on day 5 p.i. This was probably due to an unrelated infection, because control pig nasal secretions and respiratory tissues remained CA04 free for the duration of the experiment. We also measured weight gain as influenza infections reduce feed intake in pigs, which is of practical concern for swine producers. Without secondary disease, IAV infections seldom cause pigs to lose weight. In this respect, pigs are similar to humans and unlike mice that develop much more severe disease compared to natural host species as many strains express a functionally inactive form of the antiviral protein Mx1^17^,^18^. Pigs that received α-GalCer i.n., but not i.m., achieved the same body weight gain (~10% of initial weight) as Mock/Mock pigs over the challenge period (Fig. 1c). Mock/CA04 pigs did not gain any weight. These results demonstrate that α-GalCer delivered by the i.n. route can alleviate the symptoms of a severe influenza infection.

### α-GalCer administration during IAV challenge does not alter leukocyte populations in blood, lung or lymphoid tissues

**Figure 2:**
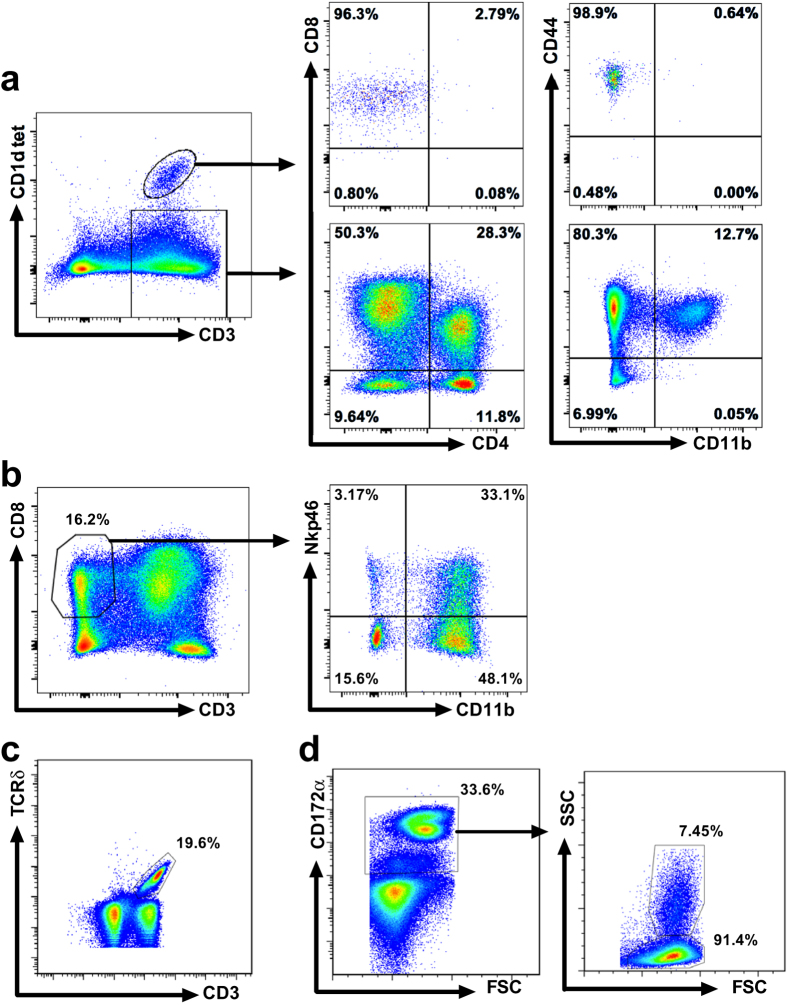
Gating strategy to identify immune cell populations in different swine tissues. Single cell suspensions were labeled with PBS57-loaded and unloaded CD1d-tetetramer and various antibodies. **(a)** T cells were identified as CD3^+^ cells after first gating on single-celled, live lymphocytes according to size and granularity. NKT-cells were distinguished from conventional T cells according to CD1d-tetramer staining. NKT-cell and conventional T cell subsets were distinguished according to surface expression of CD4, CD8α, CD44, and CD11b. **(b)** NK cells were identified as CD8α^+^ CD3^−^ cells after gating single live lymphocytes. Different NK cell subsets were distinguished from each other according to Nkp46 and CD11b surface expression **(c)** γδ T cells were identified as TCRδ^+^ CD3^+^ cells after gating on single-celled, live lymphocytes. **(d)** Monocytes and granulocytes were identified as CD172α^+^ cells with respectively high and low granularity according to side scatter. One representative lung sample is shown.

**Figure 3:**
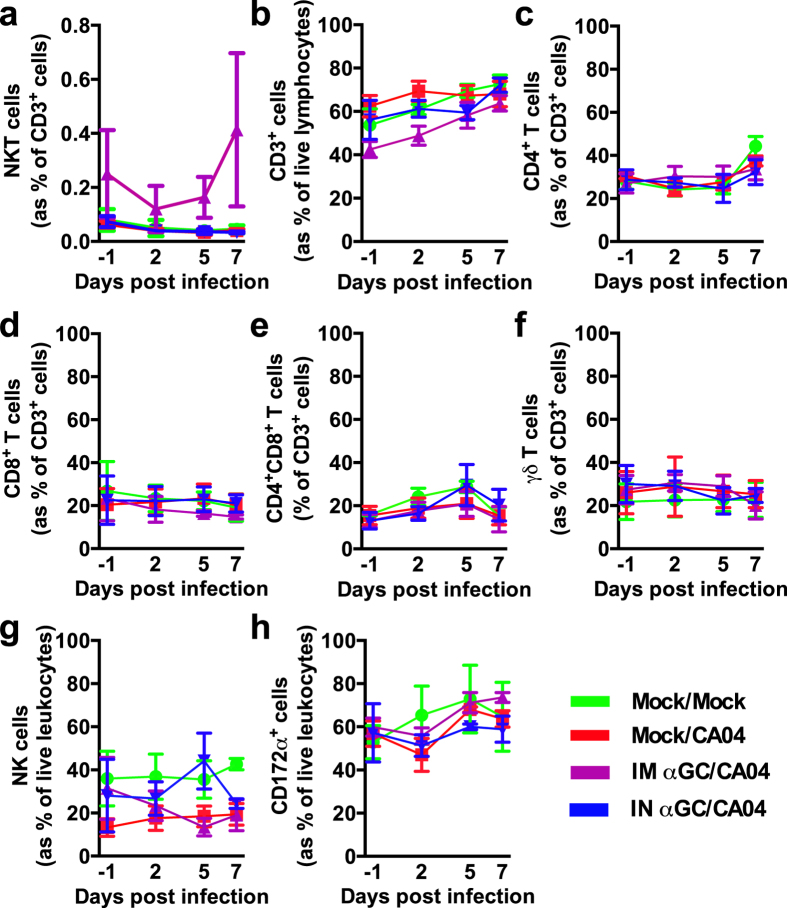
Frequency of immune cell populations analyzed from peripheral blood (PB) throughout the trial. **(a)** NKT-cells as a proportion of CD3^+^ lymphocytes, **(b)** CD3^+^ cells as a proportion of lymphocytes, **(c)** CD4^+^ single positive T cells as a proportion of CD3^+^ lymphocytes, **(d)** CD8α^+^ single positive T cells as a proportion of CD3^+^ lymphocytes, **(e)** double positive CD4^+^ CD8α^+^ T cells as a proportion of CD3^+^ lymphocytes, **(f)** γδ T cells as a proportion of CD3^+^ lymphocytes, **(g)** NK cells (CD3^−^ CD8α^+^) as a proportion of lymphocytes, **(h)** CD172α^+^ cells as a proportion of leukocytes. Differences in PB immune cell populations were analyzed using the SAS PROC MIXED procedure and no treatment differences were identified. Data are represented as mean ± SEM.

There exist important safety concerns about using α-GalCer therapy during acute influenza infections because of the pro-inflammatory responses produced by activated NKT-cells. Therefore, we evaluated the effects of IAV infection and α-GalCer administration on leukocyte populations using flow cytometry (Fig. 2) in peripheral blood (PB) throughout the challenge period (Fig. 3) and in bronchoalveolar lavage fluid (BALF) (Table 2), lung (Table 3), tracheobronchial lymph nodes (TBLN) (Supplementary Table 1) and spleen (Supplementary Table 2) after euthanasia at 7 days p.i. No differences were detected amongst treatments for NKT-cell frequency or subsets based on expression levels of CD4, CD8α, CD11b, CD335 (Nkp46) and CD44. Also, no differences were found for the frequency of αβ T cells, γδ T cells, NK cells, monocytes and granulocytes in PB and for any of the respiratory and lymphoid tissues tested. One pig in the IM αGC/CA04 group expressed particularly high levels of NKT-cells (1.6% of CD3^+^ cells in blood) compared to other pigs enrolled in the experiment. This accounts for why the average NKT-cell frequency for the IM αGC/CA04 group was relatively high throughout the study. Our results suggest that NKT-cell activation during IAV infection mitigates disease without causing changes in immune cell populations that could be potentially harmful.

### α-GalCer dramatically reduces viral replication in the respiratory tract and decreases nasal shedding

**Figure 4:**
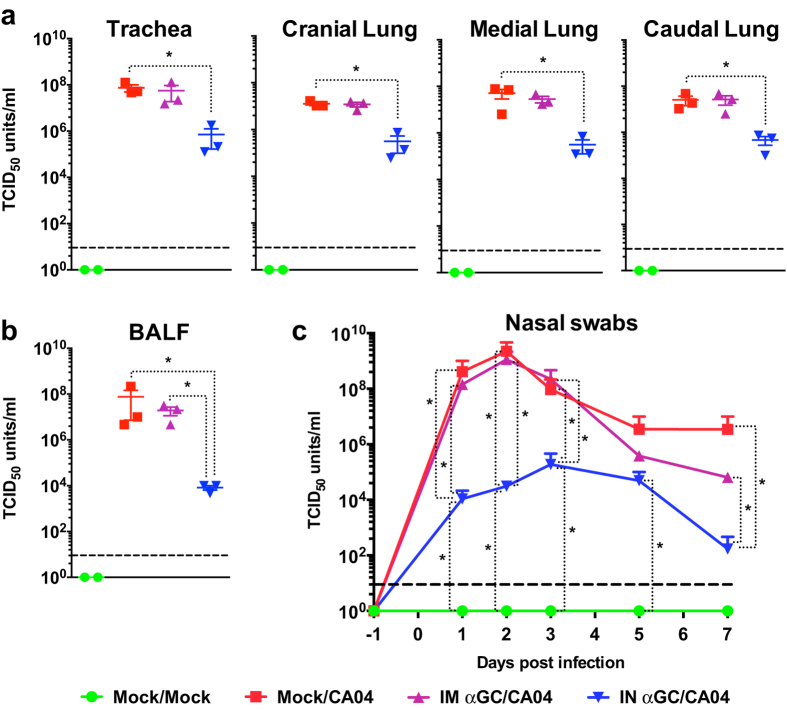
Intranasal but not intramuscular administration of α-GalCer inhibits viral replication in respiratory tissues and reduces nasal shedding in pigs challenged with CA04 virus. Viral titers for (**a**) homogenized airway tissues and (**b**) bronchoalveolar lavage fluid (BALF) collected at 7 days p.i. as well as (**c**) nasal swabs collected at −1, 1, 2, 3, 5 and 7 days p.i. Changes in nasal swab titers were analyzed using the SAS PROC MIXED procedure and the Turkey’s test was used to examine treatment differences at each time point for each dependent variable. Changes in BALF and airway tissue titers were analyzed using the Kruskal-Wallis test. *P < *0.05*. Data are represented as mean ± SEM.

We investigated the ability of α-GalCer to reduce viral replication in the airway tissues and decrease viral shedding in nasal discharge. High viral titers were detected in lung tissue and BALF of pigs in the Mock/CA04 and IM αGC/CA04 treatment groups at day 7 p.i. (Fig. 4a,b). In striking contrast, viral titers were respectively >100 fold and >9,000 fold lower in lung tissue and BALF of IN αGC/CA04 pigs. Simultaneously, nasal shedding was drastically reduced in IN αGC/CA04 pigs that had >70,000 fold less virus in nasal swabs at day 2 p.i. compared to pigs in the other virus-infected groups (Fig. 4c). Furthermore, viral titers were reduced to almost undetectable levels in IN αGC/CA04 pigs (10^2^ TCID_50_/ml) by 7 days p.i., but remained high in Mock/CA04 pigs (10^6^ TCID_50_/ml). Taken together, these results indicate that α-GalCer administration via the i.n., but not the i.m. route, generates early innate immune responses that significantly inhibits viral replication, even though piglets were infected with a high dose of CA04 virus delivered directly into the lungs.

### Activating NKT-cells during an influenza infection reduces lung pathology

**Figure 5:**
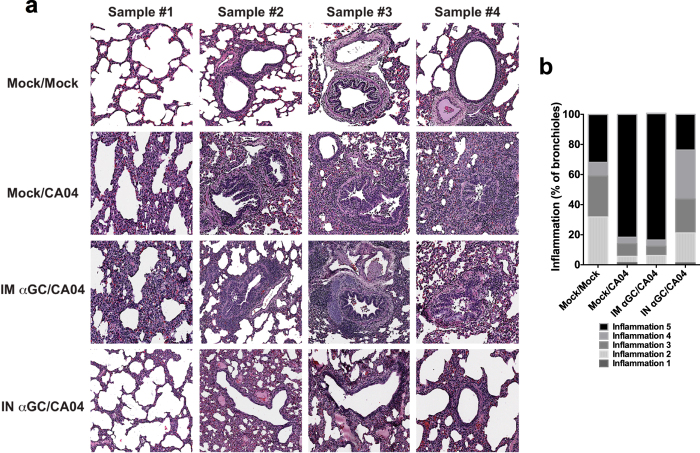
Intranasal treatment with α-GalCer reduces influenza induced lung inflammation and pathology. Lung inflammation and pathology was assessed by H&E staining on day 7 post-infection with CA04. **(a)** Four representative micrographs for the indicated treatment groups are shown. **(b)** Bronchioles in lung sections were graded by light microscopy on an arbitrary scale from 1–5 for inflammation severity; 1 = a small number of inflammatory foci and 5 = a large and widespread infiltrate, more than three cells deep. The number of bronchioles scored from each treatment was 22 for Mock/Mock, 142 for Mock/CA04, 97 for IM αGC/CA04 and 81 for IN αGC/CA04.

The decreased viral shedding and replication observed in i.n. α-GalCer treated piglets were also supported by histopathological analysis of lung sections. As shown, Mock/CA04 infected and i.m. α-GalCer treated animals developed severe pulmonary edema and peribronchiolar and perivascular inflammation (Fig. 5a,b). The degree of bronchiolar epithelial hyperplasia and airway narrowing was moderate to severe, with multiple bronchioles being affected. In contrast, lung sections from i.n. α-GalCer treated piglets had considerably fewer infiltrating cells around the bronchioles and blood vessels and relatively normal bronchial epithelium. Collectively, our data show that i.n. administration of α-GalCer dramatically reduced CA04-induced histopathological alterations in the lungs.

## Discussion

Our results show for the first time that administration of α-GalCer into the nasal passages of newly-weaned piglets significantly reduces flu symptoms and generates protective immunity that inhibits IAV replication in airway tissues, markedly decreasing viral shedding and lung pathology without any noticeable adverse effects. Our results were much better than a recent publication showing that Tamiflu, a NAI that is the leading influenza treatment for humans, has no effect on viral replication in lungs of influenza infected pigs and has little impact on nasal shedding^19^. An important advantage of NKT-cell superagonists over NAIs is that they are likely to remain an effective therapy for treating influenza even with extensive use over time. This is because activated NKT-cells provide protection that is mediated through an array of cell types and cytokines that directly and indirectly inhibit viral replication^20^. Since it is more difficult for pathogens to simultaneously develop resistance to multiple pathways than to a single pathway, the likelihood of influenza viruses becoming resistant to NKT-cell superagonists is low.

The current work also represents a first attempt to exploit NKT-cell therapy to counteract influenza in a natural host species capable of producing zoonotic infections, rather than experimental animal models. Two previous murine studies showed that α-GalCer administered by intraperitoneal (i.p.) injection increased survival in mice infected with a lethal dose of the IAV A/PR8/34^21^,^22^. A third study found that weight profile and viral titers improved in mice infected with a non-lethal dose of the human IAV E61-13-H17 when α-GalCer was administered i.p., but not through the intranasal route^23^. These reports support that α-GalCer is capable of reducing infections by different IAV strains in different genetic backgrounds. However, mice are limited for modeling IAV infections because they are generally resistant to primary human influenza isolates^24^. Conversely, when mouse-adapted influenza viruses are used, inbred mice usually develop much more severe disease compared to pigs and humans as many strains express a functionally inactive form of the antiviral protein Mx1^17^,^18^. Mice also have drawbacks for modeling NKT-cell immunotherapy because murine NKT-cell frequencies, subsets and tissue localization patterns differ considerably compared to pigs and humans^15^,^16^,^25^,^26^.

The mechanisms underlying α-GalCer-mediated influenza protection in swine remain unclear. Our study did not detect changes in the frequency of immune cells when pigs were euthanized at a late time point after challenge and treatment (7 days p.i). Uncertainty also remains about how NKT-cell agonists clear influenza infections in mice. Ho *et al*. found that protection was associated with a rapid and transient increase in chemo-attractive cytokines after α-GalCer administration followed by the influx of infiltrating neutrophils and monocytes into the lung that potentially clear virus infected epithelial cells^23^. Kok *et al*. showed that NKT-cell activation did not affect virus load but rather prevented lung injury by reducing the number of inflammatory monocytes in the respiratory tract following infection^21^. De Santo *et al*. reported that α-GalCer-activated NKT-cells decrease the accumulation of immunosuppressive myeloid-derived suppressor cells, which suppress IAV-specific immune responses in the lung through the expression of nitric oxide synthase and arginase^27^. To identify whether similar immune mechanisms contribute to protecting pigs from influenza infections, future studies should examine porcine NKT-cells and NKT-transactivated cell types within 48 hours after treatment with α-GalCer. Of interest will be whether NKT-cell-mediated IAV immunity is the same between swine and mice given that α-GalCer only protects pigs and mice when delivered via the intranasal and systemic routes, respectively. Also of practical interest will be whether NKT-cell agonists can clear influenza infections after symptoms appear.

Although additional mechanistic studies are needed, our findings provide proof-of principal that NKT-cell agonists could prove a potent strategy for improving the course of disease in swine as well as humans with seasonal and pandemic influenza infections. If successful, this approach has potential for reducing zoonotic IAV infections between swine and people that sometimes give rise to strains capable of causing pandemics. They also provide a clear rational for using NKT-cell agonists to target other microbial pathogens that threaten pig and human health.

## Methods

### Pigs

Piglets were a cross between Hampshire, Yorkshire, Chester White, Duroc, and Landrace breeds from the University of Florida swine unit. Experiments were performed in compliance with regulations from the National Research Council’s Guide for the Care and Use of Laboratory Animals, the United States Department of Agriculture and all relevant state and federal regulations and policies. The animal care and use committee at the University of Florida approved the protocol under the project number 201308209.

### Experimental design

At two-weeks of age piglets were bled from the jugular vein to measure their peripheral blood (PB) NKT-cell frequencies. Eleven piglets were selected and assigned to one of four groups so that each group contained pigs with a similar range of NKT-cell frequencies (Table 1). All animals were confirmed seronegative for antibodies against H1N1, H3N2 and B viruses by hemagglutination inhibition assays (HAI) as described^16^. At 4 weeks of age, piglets in group 1 were intratracheally sham-infected with virus-free MEM media while groups 2–4 were challenged with 10^5^ TCID_50_ CA04 as described before^16^. At the same time, piglets in groups 1–2 were administered 2 ml PBS containing 2% DMSO i.n. and piglets in groups 3–4 were administered 100 μg/kg α-GalCer dissolved in 2 ml PBS that was respectively injected into the neck muscle (i.m.) and dripped into the nostrils (i.n.). Stock solutions of α-GalCer were prepared in DMSO as previously described^15^. Control pigs were housed separately from CA04 animals.

During the 7-day challenge period, piglets were monitored daily for disease symptoms, including body weight, dyspnea, coughing, sneezing, nasal discharge, ocular discharge, eye redness and diarrhea. Nasal secretions were scored from 0 to 3, as 0: absent; 1: small volume discharge, from one nostril; 2: small volume, both nostrils; 3: large volume, both nostrils^19^,^28^. Fecal score was ranked from 0 to 3, as 0: normal; 1: semi-solid; 2: liquid; 3: watery, presence of mucous or blood^29^,^30^. Blood samples were collected at −1, 1, 2, 5 and 7 days p.i. for analysis of immune cell populations. Nasal swabs were collected at −1, 1, 2, 3, 5 and 7 days p.i. to measure viral titers. Pigs were euthanized at 7 days p.i. and tissue were collected from lung and trachea to determine viral titers. Tissue samples were also collected from lung, spleen and tracheobronchial lymph nodes (TBLN) for analysis by flow cytometry and histopathology. Bronchoalveolar lavage fluid (BALF) was collected at necropsy by lavaging the left lung with 35 ml of MEM for analysis of immune cell subsets and viral titers^16^.

### Flow cytometry and antibodies

Blood samples were collected from the jugular vein in heparinized vacutainer tubes (BD Biosciences, San Jose, CA). Spleen and lymph node tissues were dispersed into single cells as previously described^15^. Lung tissues (0.5 grams of tissue) were minced using scissors, digested with 300 μg Liberase TL (Sigma-Aldrich, Saint Louis, MO) at 37 °C for 45 minutes and filtered through 70 μm cell strainers (Falcon BD Labware, Bedford, MA). BALF was collected as described before^16^. An ammonium chloride-based lysis buffer was used to eliminate residual red blood cells from tissue samples and PB. Leukocyte populations were characterized using BD Accuri C6 and BD LSRFortessa flow cytometers (BD Bioscience) after cells were blocked and stained as previously described^16^,^31^. Staining strategies, NKT-cell tetramer reagents and antibodies used to assess NKT-cells, conventional αβ T cells, γδ T cells, monocytes and granulocytes are described in Fig. 2 and Supplemental Table 3. Data were analyzed using FlowJo software (Treestar, Palo Alto, CA).

### Virus and viral titers

Influenza virus encoding the original consensus sequence of the H1N1pdm09 strain A/California/04/2009 (CA04) was generated and recovered as previously described^5^,^16^. Viral titers were calculated from the median TCID_50_, and viral titers expressed as TCID_50_/ml, TCID_50_/gm, or their Log_10_-transformed values, as appropriate^32^,^33^. TCID_50_ values were determined by infecting MDCK cells in 96-well microtiter plates with serial dilutions of virus. Five days later the TCID_50_ was calculated according to the method of Reed and Muench^34^.

### Lung histology

At day 7 post infection, lung tissues were collected from Mock/Mock, Mock/CA04, IM α-GalCer/CA04 and IN α-GalCer/CA04 treated piglets, fixed in 10% zinc-buffered formalin, embedded in paraffin, and 5 μm thick sections were cut and stained with hematoxylin and eosin (H&E) as described before^35^. A previously described semiquantitative scoring system was used to grade the size of lung infiltrates, where 5 signified a large (more than three cells deep) widespread infiltrate around the majority of vessels and bronchioles, and 1 signified a small number of inflammatory foci^36^.

### Statistical analysis

The data were analyzed with the PROC MIXED procedure of SAS (v9.3, SAS Institute Inc., Cary, NC) when established to be normally distributed according to the QQPLOT procedures. Autoregressive covariance structure was applied to evaluate treatment effects on changes in nasal swab viral titers, after log transformation. Means were separated using Turkey’s test when a main effect or interaction term was determined to be significant (P < 0.05). Immune cell populations in blood during infection challenge were evaluated using PROC GLM of SAS. Least square means was used to determine significant difference (P < 0.05) between treatments.

Data for disease symptoms, body weight change and tissue viral titers were not normally distributed and analyzed using a nonparametric Kruscal-Wallis test with GraphPad Prism (version 6.0 h for Macintosh, GraphPad Software Inc., La Jolla, CA). Means were separated using Dunn’s multiple comparisons test when a main effect or interaction term was determined to be significant (P < 0.05). Linear regression analyses were preformed using GraphPad Prism.

## Additional Information

**How to cite this article**: Artiaga, B. L. *et al*. Rapid Control of Pandemic H1N1 Influenza by Targeting NKT-cells. *Sci. Rep.* **6**, 37999; doi: 10.1038/srep37999 (2016).

**Publisher's note:** Springer Nature remains neutral with regard to jurisdictional claims in published maps and institutional affiliations.

## Supplementary Material

Supplementary Material

## Figures and Tables

**Table 1:** Characteristics of pigs enrolled in the experiment.

Treatment groups	Number of animals	Initial NKT-cell frequency (% of CD3^+^ cells)	Initial body weight (kg)
Mock/Mock	2	0.15 ± 0.01	5.40 ± 1.70
Mock/CA04	3	0.27 ± 0.06	5.27 ± 0.45
IM αGC/CA04	3	0.67 ± 0.46	4.90 ± 0.60
IN αGC/CA04	3	0.26 ± 0.03	4.97 ± 0.66

Values represent mean ± SEM. Initial NKT-cell frequency was analyzed from peripheral blood at 2 weeks of age. Initial body weight was recorded one day before treatment.

**Table 2:** Frequency of leukocyte populations in bronchoalveolar lavage fluid (BALF) at day 7 post infection.

Immune cell population	Mock/Mock	Mock/CA04	IM αGC/CA04	IN αGC/CA04
CD1d tetramer^+^ NKT cells (of CD3^+^)	0.08 ± 0.01	0.12 ± 0.01	0.49 ± 0.27	0.07 ± 0.03
CD8α^+^ (of CD3^+^)	81.10 ± 1.00	85.60 ± 1.31	84.03 ± 4.40	84.33 ± 1.17
CD4^+^ (of CD3^+^)	4.63 ± 0.09	2.23 ± 0.66	2.62 ± 0.53	3.85 ± 1.87
CD4^+^CD8α^+^ (of CD3^+^)	9.30 ± 0.69	6.29 ± 0.91	8.99 ± 2.99	7.22 ± 1.22
CD3^-^CD8α^+^ NK cells (of lymphocytes)	5.67 ± 0.84	7.47 ± 2.51	7.54 ± 1.58	8.34 ± 2.33
Granulocytes (FSC^hi^ SSC^hi^ of live)	72.4 ± 13.8	82.8 ± 5.52	78.9 ± 10.21	77.9 ± 5.21

Values represent mean ± SEM for lymphocyte-sized single cells and cell population indicated in parenthesis. No significant difference (p > 0.05) was detected between treatments for any of the immune cell populations tested when analyzed by the Kruskal-Wallis test. Mock treated and mock challenged (Mock/Mock); mock treated and CA04 challenged (Mock/CA04); 100 μg/kg α**-**GalCer administered i.m. (IM αGC/CA04) or i.n. (IN αGC/CA04).

**Table 3:** Frequency of leukocyte populations in lung tissue at day 7 post infection.

Immune cell population	Mock/Mock	Mock/CA04	IM αGC/CA04	IN αGC/CA04
CD1d tetramer^+^ NKT cells (of CD3^+^)	0.10 ± 0.02	0.12 ± 0.02	0.93 ± 0.69	0.17 ± 0.06
CD8α^+^ (of CD3^+^)	64.05 ± 13.55	71.70 ± 8.18	69.73 ± 6.26	61.90 ± 7.18
CD4^+^ (of CD3^+^)	7.02 ± 3.09	5.81 ± 3.90	6.66 ± 2.60	8.10 ± 3.67
CD4^+^CD8α^+^ (of CD3^+^)	21.05 ± 5.95	15.67 ± 1.31	17.53 ± 4.55	21.53 ± 4.77
CD3^-^CD8α^+^ NK cells (of lymphocytes)	12.30 ± 5.41	6.79 ± 1.04	10.97 ± 0.87	15.57 ± 0.35
Monocytes (CD172α^+^SSC^low^ of live)	1.78 ± 0.31	1.89 ± 1.03	1.89 ± 0.56	1.73 ± 0.29
Granulocytes (CD172α^+^ SSC^hi^ of live)	18.15 ± 3.25	11.29 ± 2.25	14.23 ± 2.61	10.56 ± 0.53

Values represent mean ± SEM for lymphocyte-sized single cells and cell population indicated in parenthesis. No significant difference (p > 0.05) was detected between treatments for any of the immune cell populations tested when analyzed by the Kruskal-Wallis test. Mock treated and mock challenged (Mock/Mock); mock treated and CA04 challenged (Mock/CA04); 100 μg/kg α-GalCer administered i.m. (IM αGC/CA04) or i.n. (IN αGC/CA04).
