# Supplementary material for: Rapid control of pandemic H1N1 influenza by targeting NKT-cells
Source: Sci Rep. 2016 Nov 29;6:37999. doi: 10.1038/srep37999 (PMC5126553; doi:10.1038/srep37999)
Supplement: Supplementary Material [file srep37999-s1.pdf]

Supplementary Information

Rapid control of pandemic H1N1 influenza by targeting NKT-cells

Bianca L. Artiaga[#1], Guan Yang[#1], Tarun E. Hutchinson[2], Julia C. Loeb[3], Jürgen A. Richt[4], John A. Lednicky[3,5], Shahram Salek-Ardakani[2], and John P. Driver*[1]

[1]Department of Animal Science, University of Florida, Gainesville, FL, USA;

[2]Department of Pathology, Immunology, and Laboratory Medicine, University of Florida, Gainesville, FL, USA;

[3]Emerging Pathogens Institute, University of Florida, Gainesville, FL, USA;

[4]Diagnostic Medicine and Pathobiology and Center of Excellence for Emerging and Zoonotic Animal Diseases (CEEZAD), College of Veterinary Medicine, Kansas State University, Manhattan, KS, USA;

[5]Department of Environmental and Global Health, University of Florida, Gainesville, FL, USA.

*Correspondence: John P. Driver

jdriver@ufl.edu

[#]B.L.A. and G.Y. contributed equally to this work.

Supplemental Table 1. Frequency of leukocyte populations in tracheobronchial lymph nodes (TBLN) at day 7 post infection

Immune cell population	Mock/Mock	Mock/CA04	IM αGC/CA04	IN αGC/CA04
CD1d tetramer$^+$ NKT cells (of CD3$^+$)	0.19 ± 0.06	0.06 ± 0.01	0.23 ± 0.10	0.06 ± 0.01
CD8α$^+$ (of CD3$^+$)	25.35 ± 5.45	21.57 ± 2.14	21.43 ± 2.54	23.43 ± 2.58
CD4$^+$ (of CD3$^+$)	22.40 ± 3.90	18.47 ± 8.63	28.47 ± 9.75	28.60 ± 9.11
CD4$^+$CD8α$^+$ (of CD3$^+$)	48.85 ± 10.35	55.60 ± 11.70	45.10 ± 13.81	44.10 ± 12.90
CD3$^-$CD8α$^+$ NK cells (of lymphocytes)	3.55 ± 0.35	8.93 ± 1.15	5.15 ± 0.78	8.07 ± 1.65
Granulocytes (FSC^{hi} SSC^{hi} of live)	0.63 ± 0.46	1.13 ± 0.33	0.42 ± 0.02	0.47 ± 0.19

Values represent mean ± SEM for lymphocyte-sized single cells and cell population indicated in parenthesis. No significant difference ($p > 0.05$) was detected between treatments for any of the immune cell populations tested when analyzed by the Kruskal-Wallis test. Mock treated and mock challenged (Mock/Mock); mock treated and CA04 challenged (Mock/CA04); 100 μg/kg α-GalCer administered i.m. (IM αGC/CA04) or i.n. (IN αGC/CA04).

Supplemental Table 2. Frequency of leukocyte populations in spleen at day 7 post infection

Immune cell population	Mock/Mock	Mock/CA04	IM αGC/CA04	IN αGC/CA04
CD1d tetramer$^+$ NKT cells (of CD3$^+$)	0.39 ± 0.00	0.32 ± 0.07	1.82 ± 1.02	0.58 ± 0.22
CD8α$^+$ (of CD3$^+$)	56.65 ± 9.15	52.80 ± 5.09	51.33 ± 5.44	48.17 ± 1.44
CD4$^+$ (of CD3$^+$)	7.63 ± 6.07	7.97 ± 2.45	7.34 ± 3.73	8.57 ± 3.03
CD4$^+$CD8α$^+$ (of CD3$^+$)	25.50 ± 1.00	22.47 ± 4.56	29.37 ± 2.11	30.60 ± 6.45
CD3$^-$CD8α$^+$ NK cells (of lymphocytes)	9.75 ± 0.85	8.15 ± 0.52	7.74 ± 1.44	7.53 ±2.44
TCRδ$^+$ γδ T cells (of CD3$^+$)	29.60 ± 2.00	38.37 ± 4.35	29.97 ± 3.21	35.73 ± 4.89
Monocytes (CD172α$^+$SSC^{low} of live)	4.06 ± 1.69	3.98 ± 0.26	4.98 ± 0.28	3.43 ± 0.22
Granulocytes (CD172α$^+$SSC^{hi} of live)	7.27 ± 2.83	7.72 ± 1.93	9.92 ± 0.35	8.20 ± 3.05

Values represent mean ± SEM for lymphocyte-sized single cells and cell population indicated in parenthesis. No significant difference ($p > 0.05$) was detected between treatments for any of the immune cell populations tested when analyzed by the Kruskal-Wallis test. Mock treated and mock challenged (Mock/Mock); mock treated and CA04 challenged (Mock/CA04); 100 μg/kg α-GalCer administered i.m. (IM αGC/CA04) or i.n. (IN αGC/CA04).

Supplemental Table 3. Reagents used for flow cytometry analysis of surface markers

Antigen	Clone	Isotype	Fluorochrome conjugation	Source
CD1d tetramer	N/A	N/A	PE	NIH Tetramer Core Facility
Anti-CD3e	BB23-8E6-8C8	Mouse IgG2a k	FITC/PE/PeCy7	BD Biosciences
Anti-CD8α	76-2-11	Mouse IgG2a k	PE/Biotin	BD Biosciences
Anti-CD4	74-12-4	Mouse IgG2b k	Alexa647	Southern Biotech
Anti-TCRd	PGBL22A	Mouse IgG1	Alexa647	Washington State University
Anti-CD172α	74-22-15A	Mouse IgG2b k	Alexa488	BD Biosciences
Anti-CD11b	M1/70	Rat IgG2b k	BV421	BioLegend
Anti-CD44	IM7	Rat IgG2b k	BV650	BioLegend
Anti-CD335 (Nkp46)	VIV-KM1	Mouse IgG1	Alexa488	AbD Serotec
